# Supplementary material for: ProteinShader: illustrative rendering of macromolecules
Source: BMC Struct Biol. 2009 Mar 30;9:19. doi: 10.1186/1472-6807-9-19 (PMC2672931; doi:10.1186/1472-6807-9-19)
Supplement: Additional file 1 — ProteinShader program without source code. This compressed file contains the complete ProteinShader program including associated libraries, but no source code. A README.txt file gives an overview of the ProteinShader distribution, and the index.html file in the help subdirectory has directions on getting started with the program as well as a set of tutorials. [file 1472-6807-9-19-S1.zip › ProteinShader-beta-0_9_4-binary/help/api/org/proteinshader/structure/enums/ResidueEnum.html]

ResidueEnum (ProteinShader API)


|  |  |  |
| --- | --- | --- |
| **Overview** \| **Package** \| **Class** \| **Use** \| **Tree** \| **Deprecated** \| **Index** \| **Help** | |  |
| **PREV CLASS**   **NEXT CLASS** | **FRAMES**    **NO FRAMES**     **All Classes** |  |
| SUMMARY: NESTED \| ENUM CONSTANTS \| FIELD \| METHOD | DETAIL: ENUM CONSTANTS \| FIELD \| METHOD |  |


---


## org.proteinshader.structure.enums Enum ResidueEnum

```
java.lang.Object
  java.lang.Enum<ResidueEnum>
      org.proteinshader.structure.enums.ResidueEnum
```

**All Implemented Interfaces:**: Serializable, Comparable<ResidueEnum>

---

``` public enum ResidueEnum extends Enum<ResidueEnum> ```

Provides an enumeration that can be used to restrict a Visitor to
traversing only a particular Residue type.

---

**Enum Constant Summary**

`AA_AND_WATERS`
  
          Visit AminoAcids first and then visit Waters.

`AA_BACKBONE`
  
          Visit only AminoAcids, and then visit
only the backbone Atoms.

`AA_SIDE_CHAIN`
  
          Visit only AminoAcids, and then visit
only the side chain Atoms.

`AMINO_ACIDS`
  
          Visit only AminoAcids.

`HETEROGENS`
  
          Visit only Heterogens.

`WATERS`
  
          Visit only Waters.


| **Method Summary** | |
| --- | --- |
| `String` | `toString()`             Returns the Residue type as a String suitable for use in a menu: for example, "Amino Acids" rather then "AMINO\_ACIDS". |
| `static ResidueEnum` | `valueOf(String name)`             Returns the enum constant of this type with the specified name. |
| `static ResidueEnum[]` | `values()`             Returns an array containing the constants of this enum type, in the order they're declared. |

**Methods inherited from class java.lang.Enum**

`clone, compareTo, equals, getDeclaringClass, hashCode, name, ordinal, valueOf`

**Methods inherited from class java.lang.Object**

`finalize, getClass, notify, notifyAll, wait, wait, wait`

**Enum Constant Detail**

### AMINO\_ACIDS

```
public static final ResidueEnum AMINO_ACIDS
```

:   Visit only AminoAcids.

---


### AA\_BACKBONE

```
public static final ResidueEnum AA_BACKBONE
```

:   Visit only AminoAcids, and then visit
    only the backbone Atoms.

---


### AA\_SIDE\_CHAIN

```
public static final ResidueEnum AA_SIDE_CHAIN
```

:   Visit only AminoAcids, and then visit
    only the side chain Atoms.

---


### AA\_AND\_WATERS

```
public static final ResidueEnum AA_AND_WATERS
```

:   Visit AminoAcids first and then visit Waters.

---


### HETEROGENS

```
public static final ResidueEnum HETEROGENS
```

:   Visit only Heterogens.

---


### WATERS

```
public static final ResidueEnum WATERS
```

:   Visit only Waters.


**Method Detail**

### values

```
public static final ResidueEnum[] values()
```

:   Returns an array containing the constants of this enum type, in
    the order they're declared. This method may be used to iterate
    over the constants as follows:

    ```
    for(ResidueEnum c : ResidueEnum.values())
            System.out.println(c);
    ```

    :   **Returns:**: an array containing the constants of this enum type, in the order they're declared

---


### valueOf

```
public static ResidueEnum valueOf(String name)
```

:   Returns the enum constant of this type with the specified name.
    The string must match *exactly* an identifier used to declare an
    enum constant in this type. (Extraneous whitespace characters are
    not permitted.)

    :   **Parameters:**: `name` - the name of the enum constant to be returned. **Returns:**: the enum constant with the specified name **Throws:**: `IllegalArgumentException` - if this enum type has no constant with the specified name

---


### toString

```
public String toString()
```

:   Returns the Residue type as a String suitable for use in a menu:
    for example, "Amino Acids" rather then "AMINO\_ACIDS".

    :   **Overrides:**: `toString` in class `Enum<ResidueEnum>`
    :   **Returns:**: The Residue type as a String.


---


|  |  |  |
| --- | --- | --- |
| **Overview** \| **Package** \| **Class** \| **Use** \| **Tree** \| **Deprecated** \| **Index** \| **Help** | |  |
| **PREV CLASS**   **NEXT CLASS** | **FRAMES**    **NO FRAMES**     **All Classes** |  |
| SUMMARY: NESTED \| ENUM CONSTANTS \| FIELD \| METHOD | DETAIL: ENUM CONSTANTS \| FIELD \| METHOD |  |


---

# *Copyright © 2007-2008*
